# The current status of health literacy among caregivers of lung cancer patients:A cross-sectional survey

**DOI:** 10.1371/journal.pone.0345901

**Published:** 2026-07-23

**Authors:** Lei Lei, Yuan Jiang, Gang Feng, Jing Li, Danfeng Wu, Xiaoyan Wang, Xiaojing Xue

**Affiliations:** 1 Department of Oncology, Mianyang Central Hospital Affiliated Hospital of 2 Electronic Science and Technology University, Mianyang, China; 2 Department of Nursing, Mianyang Central Hospital Affiliated Hospital of Electronic Science and Technology University, Mianyang, China; Sichuan University, CHINA

## Abstract

**Aim:**

This exploratory, hypothesis-generating study aimed to investigate the current status of cancer health literacy among lung cancer caregivers and to identify factors associated with their health literacy levels. The primary hypothesis was that caregivers would demonstrate sub optimal cancer health literacy. The primary endpoint was the total score on the Chinese Cancer Health Literacy Scale (C-CHLS). Secondary endpoints included scores on the Health Literacy of Caregivers Scale-Cancer (HLCS-C) and the identification of factors associated with caregivers’ health literacy.

**Background:**

Health literacy, defined as an individual’s ability to acquire, comprehend, and utilize health-related information to make informed decisions for maintaining health, is a critical factor in cancer prevention and treatment. It significantly impacts individual health outcomes.While existing research primarily focuses on cancer patients,there is limited exploration into the health literacy of cancer caregivers, who play a vital role in patient support and care.

**Design:**

A cross-sectional study.

**Methods:**

Using convenience sampling, 198 caregivers of lung cancer patients receiving treatment at a tertiary hospital in Sichuan Province, China, from October 2024 to December 2024 were recruited. Data were collected using a general information questionnaire,health literacy of caregivers scale-cancer and Chinese cancer prevention and control literacy questionnaire. Data entry was performed using Microsoft Excel 2016, and statistical analysis was conducted using SPSS 26.0.

**Results:**

Only 48.48% (n = 96) of caregivers demonstrated adequate health literacy. Multivariable analysis identified seven factors significantly associated with cancer health literacy (all *p* < 0.05): younger age (β = 0.187, t = 3.189), higher education level (β = 0.209, t = 2.784), professional occupation (β = 0.169, t = 2.144), better self-rated health (β = 0.186, t = 3.091), non-smoking status (β = 0.175, t = 2.514), non-alcohol consumption (β = 0.163, t = 2.412), and higher perceived cancer risk (β = 0.286, t = 4.274).

**Conclusion:**

The suboptimal health literacy levels among lung cancer caregivers observed in this sample underscore the potential need for tailored educational interventions. Future programs may consider addressing modifiable factors, particularly focusing on older, less-educated caregivers with health-risk behaviors. These findings provide preliminary evidence for developing targeted strategies to improve caregiver health literacy in similar clinical settings.

## 1. Introduction

Lung cancer remains one of the most formidable public health challenges worldwide, with profound implications for morbidity, mortality, and healthcare systems. According to the latest estimates from the International Agency for Research on Cancer (IARC), approximately 2.2 million new cases of lung cancer were diagnosed globally in 2022, making it the second most commonly occurring cancer after breast cancer but the leading cause of cancer-related deaths, responsible for nearly 1.8 million fatalities annually [1]. The high mortality rate is attributed to late-stage diagnosis, aggressive tumor biology, and limited treatment options for advanced disease.

In China, the situation is particularly alarming. Lung cancer constitutes 23.8% of all cancer-related deaths, reflecting its disproportionate burden on the population [2]. The rising incidence is driven by a combination of factors, including tobacco use, air pollution, occupational exposures and indoor pollutants such as cooking fumes in poorly ventilated homes [3]. Moreover, the five-year survival rate for lung cancer in China remains below 20%, significantly lower than in high-income countries, partly due to delayed detection and limited access to advanced therapies in rural areas [4].

Lung cancer patients often experience prolonged illness trajectories, marked by debilitating symptoms such as dyspnea, chronic cough, fatigue, and pain, as well as comorbidities like chronic obstructive pulmonary disease (COPD) or cardiovascular disease [5]. Given the high caregiving demands, family members(primarily spouses and adult children)serve as informal yet critical healthcare providers, assuming responsibilities that include:symptom monitoring and management,medication adherence support,emotional and psychological support and medical decision-making [6].

Research underscores that caregivers’ health literacy—their ability to access, understand, and apply health information---directly impacts patient outcomes [7]. For example, caregivers with limited symptom management skills may inadvertently increase patients’ risk of hospital readmission by 30% due to unrecognized complications like infections or treatment toxicities [8]. Conversely, high caregiver health literacy correlates with improved patient quality of life, treatment adherence, and even survival rates [9].

Despite their pivotal role, caregivers frequently neglect their own health, prioritizing outpatients over their own. Chronic stress and burnout are prevalent among caregivers, with studies showing elevated risks for depression, insomnia, and cardiovascular diseases [10]. Also,Caregivers often forgo routine cancer screenings or lifestyle modifications, despite their increased risk due to shared environmental or behavioral factors [11].

Health literacy is a modifiable determinant of health behaviors and outcomes. The WHO defines it as the cognitive and social skills enabling individuals to access, process, and act on health information [12]. In oncology, higher health literacy is associated with:Earlier cancer detection,Better prevention practices and Enhanced treatment adherence.Internationally, cancer prevention and control literacy (CPCL) frameworks have been developed, encompassing:knowledge,Skills and behavior [13]. While Western studies have robust CPCL frameworks, research in China remains patient-Centric, with scant attention to caregivers. Key limitations include:Tool adaptation(Most studies use non-validated translations of foreign scales, lacking cultural relevance) and Limited evidence(Few surveys assess lung cancer caregivers, and none systematically examine socioeconomic, educational, or regional disparities in their health literacy).

This cross-sectional study systematically evaluates cancer prevention and treatment literacy among lung cancer caregivers in China—an underexplored yet critical population. Adopting a caregiver-centered approach, it employs culturally adapted tools (C-CHLS and HLCS-C) to capture multidimensional health literacy.Exploratory and hypothesis-generating in nature, the study posits that caregivers’ cancer health literacy is suboptimal. The primary endpoint is the C-CHLS total score, measuring basic cancer prevention and control literacy. Secondary endpoints include HLCS-C scores, which assess multidimensional health literacy, and factors associated with caregivers’ literacy levels.By identifying key modifiable factors associated with caregivers’ health literacy, this study provides evidence to guide targeted educational interventions. Findings aim to inform clinical guidelines and public health strategies, ultimately enhancing caregiver capacity and patient care quality while supporting global efforts to reduce the lung cancer burden.

## 2. Methods

### 2.1. Settings and patients

We conducted a prospective survey study of primary caregivers for lung cancer patients admitted to the Department of Oncology at a tertiary care center in Sichuan Province, China, between October and December 2024. A convenience sampling method was used to recruit participants. All investigators who participated in this study provided written informed consent.Inclusion criteria required participants to: (1) be aged ≥18 years; (2) serve as the primary caregiver for a lung cancer patient; and (3) demonstrate adequate cognitive and comprehension abilities as assessed by trained research staff. We excluded individuals with: (1) physician-diagnosed mental health disorders (e.g., major depressive disorder, schizophrenia); (2) documented cognitive impairment (Mini-Mental State Examination score <24); or (3) exposure to major stressful life events (e.g., bereavement, job loss) within 30 days prior to enrollment.

### 2.2. Instruments

#### 2.2.1 General information questionnaire.

The General Information Questionnaire was designed to collect sociodemographic characteristics and disease-related data. Sociodemographic variables encompassed variables such as age, gender, ethnicity, education level, occupation, marital status, family size, per capita monthly income, and medical insurance status. Disease-related variables included smoking history, alcohol consumption, body mass index (BMI), self-rated overall health status, family history of cancer, and the specific type of tumor diagnosed in the patient.

#### 2.2.2 The Chinese Cancer Health Literacy Scale (C-CHLS).

The Chinese Cancer Health Literacy Scale (C-CHLS), developed by the National Cancer Center of China [14], is a validated assessment tool comprising 24 core items with 40 sub-items. The instrument evaluates five critical domains of cancer-related health literacy: (1)prevention awareness, (2) early detection knowledge, (3) diagnostic understanding, (4) treatment awareness, and (5) information-seeking preferences.The total score ranges from 0 to 40 points, with a validated threshold of ≥15.2 points (38% of maximum score) indicating adequate cancer health literacy (sensitivity: 82.3%; specificity: 76.5%). In this study, the C-CHLS total score served as the primary endpoint, as it directly measures caregivers’ basic cancer prevention and control literacy, which aligns with the primary hypothesis that such literacy is suboptimal.

#### 2.2.3 Health Literacy of Caregivers Scale-Cancer (HLCS-C).

The HLCS-C, originally developed and validated by Yuen et al. [15], is a multidimensional instrument comprising 46 items organized into 10 clinically relevant domains: (1) information-seeking motivation, (2) cancer-specific knowledge, (3)clinician-assisted comprehension, (4) social support utilization, (5) patient-provider communication, (6) patient-centered care understanding, (7) self-care capacity, (8) healthcare system navigation, (9) medical information processing, and (10) proactive clinical engagement.

The total score ranges from 45 to 188 points, with established classification thresholds:45–93 indicating Low health literacy,94−140 indicating Moderate health literacy,141−188 indicating High health literacyPsychometric evaluation demonstrated strong internal consistency across all subscales (Cronbach’s α range: 0.78–0.92), indicating excellent reliability for clinical and research applications. In this study, HLCS-C scores served as secondary endpoints, providing a multidimensional assessment of caregivers’ health literacy and enabling exploration of factors associated with different domains.

### 2.3. Data collection

Potential caregiver participants were systematically screened against predetermined eligibility criteria. Following informed consent procedures, both patients and their caregivers received comprehensive explanations regarding: (1) study objectives, (2) scientific significance, (3) methodological approach, (4) anticipated time commitment, and (5) relevant participation considerations. Written confidentiality agreements ensured all collected personal and clinical data would remain strictly confidential and be utilized solely for research purposes.

Standardized questionnaire administration occurred 24 ± 2 hours post-admission to minimize acute hospitalization stress bias. The protocol mandated independent completion by caregivers under normal circumstances. For participants with literacy challenges, certified research assistants provided standardized, non-directive question clarification following a predetermined script, with verbatim response documentation to maintain data integrity.

### 2.4 Ethical considerations

All methods were performed in accordance with the relevant guidelines and regulations or in accordance with the Declaration of Helsinki. All participants or their

families provided informed consent to participate.

This clinical study has been approved by the Ethics Committee on Biomedical Research, Mianyang Central Hospital Affiliated Hospital of Electronic Science and Technology University (File No.S202403109-02).

### 2.5. Data analysis

Data entry was performed using Microsoft Excel 2016, and statistical analysis was conducted using SPSS 26.0. Descriptive statistics were presented as frequencies and percentages for categorical variables, mean ± standard deviation (SD) for normally distributed continuous variables, and quartiles for non‑normally distributed continuous variables. Pearson or Spearman correlation coefficients were used to assess associations between continuous variables, as appropriate. Univariate analyses comparing health literacy scores across categorical variables were performed using independent samples t‑test or one‑way ANOVA.Given the exploratory nature of these analyses, results were interpreted without formal adjustment for multiple comparisons, and findings should be considered hypothesis-generating. Multivariable linear regression was employed to identify factors independently associated with caregivers’ cancer health literacy. A p-value of less than 0.05 was considered statistically significant.

## 3. Results

This study enrolled 198 primary caregivers of lung cancer patients (mean age = 48.06 ± 14.60 years; range:18–82 years), with a balanced gender distribution (males:42.42%, n = 84; females:57.58%, n = 114). Age distribution followed a normal pattern, with the largest proportion (47.48%, n = 94) being middle-aged adults (40–59 years).

In terms of their relationship to the patient, spouses represented the largest group (38.39%, 76 cases), followed by children (30.81%, 61 cases), parents (13.13%, 26 cases), and grandchildren or other relatives (17.17%, 34 cases). Regarding education level, the majority of caregivers had completed high school (23.23%, 46 cases) or held an undergraduate degree or higher (33.84%, 67 cases). Most caregivers were married (82.32%, 163 cases).

In terms of income, 37.37% (74 caregivers) reported a monthly income per capita of less than 2,101 yuan, while 32.33% (64 caregivers) had a monthly income between 2,101 and 5,000 yuan. The most common type of medical insurance was urban and rural resident medical insurance, covering 58.08% (115 caregivers).

The body mass index (BMI) of most caregivers was within the normal range (18.5–23.9 kg/m²), with 53.54% (106 caregivers) meeting this criterion. When asked to rate their health status over the past week, most caregivers described it as “good” (61.11%, 121 cases). For further details, refer to Table 1.

**Table 1:** General demographic, socioeconomic, and health-related characteristics of lung cancer caregivers (n = 198).

Variables	n (%)	Variables	n(%)
Gender		Ethnic group	
male	84(42.42)	Han	196(98.99)
female	114(57.58)	Qiang	2(1.01)
Age		Per capita monthly income of family	
<40	66(33.33)	<2101	74(37.37)
40-59	94(47.48)	2101-5000	64(32.33)
≥ 60	38(19.19)	>5000	60(30.30)
Degree of education		Occupation	
Bachelor degree	67(33.84)	staff	165(83.33)
High school diploma	46(23.23)	farmer	33(16.67)
Middle school diploma	37(18.69)	Medical insurance	
Primary school diploma	48(24.24)	Urban medical insurance	82(41.41)
Marriage		Rural medical insurance	115(58.08)
single	35(17.68)	others	1(0.01)
married	163(82.32)	health self-assessment	
BMI		bad	17(38.89)
<18.5	9(4.54)	normal	60(30.30)
18.5-23.9	106(53.54)	good	121(61.11)
≥24	83(41.92)		

### 3.1. General information of the research subjects

### 3.2. Descriptive analysis of cancer health literacy among caregivers

The total and dimensional scores of cancer health literacy demonstrated normal distribution (Kolmogorov-Smirnov test, all P > 0.05), expressed as mean±SD. Using the validated cutoff (≥15.2 points), 48.48% (n = 96/198) of caregivers demonstrated adequate cancer health literacy. The population’s mean score (14.59 ± 3.29 points) significantly fell below this threshold (t = 6.142, *P* < 0.001), indicating suboptimal literacy levels.

In a survey assessing current knowledge of cancer prevention and control, caregivers identified the top three most prevalent cancers in China as lung cancer (78.79%), stomach cancer (67.68%), and liver cancer (63.13%). Additionally, 173 caregivers (87.37%) perceived an increase in the number of cancer cases in China and ranked the top three risk factors for cancer as smoking and alcohol consumption (83.84%), unhealthy dietary habits (79.29%), and environmental pollution (64.14%).

Regarding preventive behaviors, 113 caregivers (57.07%) reported regularly consuming fresh fruits and vegetables to reduce cancer risk. Furthermore, 181 caregivers (91.41%) and 156 caregivers (78.79%) recognized that consuming moldy grains and contaminated pond water, respectively, could increase the risk of cancer. For further details, refer to Table 2.

**Table 2:** Awareness of common cancers, risk factors, and preventive behaviors among lung cancer caregivers (n = 198).

Variables	n(%)	Variables	n(%)
The most common type of cancer in China		Inducing factors of cancer	
lung cancer	156(78.79)	Smoking and drinking	166(83.84)
stomach cancer	134(67.68)	infection	100(50.51)
hepatocarcinoma	125(63.13)	Unreasonable diet	157(79.29)
Carcinoma of esophagus	108(54.55)	Occupational hazard	99(50.0)
breast carcinoma	109(55.05)	Environmental pollution	127(64.14)
cervical cancer	90(45.45)	Above of all	57(28.79)
carcinoma of the rectum	83(41.92)	unclear	11(5.56)
carcinoma of colon	62(31.31)	Measures to reduce the incidence of cancer	
Number of cancer patients in China		Stop smoking and drinking	184(92.93)
more than before	173(87.37)	Injecting hepatitis B vaccine	88(44.44)
on the decrease	2(1.01)	Balance diet and exercise	176(88.89)
unclear	23(11.62)	Eliminating occupational hazards	98(49.49)
Do you think eating fresh fruits and vegetables regularly can prevent cancer?		Above of all	64(32.32)
Yes	113(57.07)	Do you think drinking unclean water regularly will lead to cancer?	
No	24(12.12)	Yes	156(78.79)
unclear	61(30.81)	No	4(2.02)
Do you think eating moldy food will lead to cancer?		unclear	38(19.19)
Yes	181(91.41)		
No	1(0.51)		
unclear	16(8.08)		

### 3.3. Description of cancer health literacy dimensions among caregivers

This study assessed the cancer health literacy of 198 caregivers of lung cancer patients across several dimensions, including awareness of cancer prevention, early detection, early diagnosis, early treatment, knowledge needs for tumor prevention, and overall cancer prevention literacy. The average scores for these dimensions were as follows: cancer prevention awareness (7.60 ± 1.66 points), early detection (2.05 ± 0.98 points), early diagnosis (1.75 ± 0.55 points), early treatment (1.83 ± 0.56 points), tumor prevention knowledge needs (1.37 ± 0.75 points), and overall cancer prevention literacy (14.59 ± 3.29 points).

Among the caregivers, only 96 (48.48%) achieved a total score of ≥ 15.2 points, indicating basic literacy in cancer prevention and control. For further details, refer to Table 3.

**Table 3:** Scores of cancer prevention and treatment literacy dimensions among lung cancer caregivers (n = 198).

Dimension	n(%)	Score
Prevention awareness score(10 points in total)		7.60 ± 1.66
Early detection awareness score (3 points in total)		2.05 ± 0.98
Early diagnostic awareness score (2 points in total)		1.75 ± 0.55
Early treatment awareness score (2 points in total)		1.83 ± 0.56
cancer prevention and treatment knowledge demand score (2 points in total)		1.37 ± 0.75
Total score		14.59 ± 3.29
have basic cancer health literacy		
Yes	96(48.48)	
No	102(51.52)	

### 3.4. Description of Health literacy score of caregivers

The total score of health literacy of caregivers of patients with lung cancer was (129.46 ± 17.42 points), and the average scores of each dimension are shown in Table 4.

**Table 4:** Scores of health literacy dimensions (HLCS-C) among lung cancer caregivers (n = 198).

Dimension	Score
Dimension 1 Proactivity and determination to seek information	2.70 ± 0.77
Dimension 2 Adequate information about cancer and cancer management	2.60 ± 0.59
Dimension 3 Supported by HCPs to understand information	3.02 ± 0.43
Dimension 4 Social support	3.14 ± 0.53
Dimension 5 Cancer-related communication with the care recipient	2.80 ± 0.72
Dimension 6 Understanding care recipient needs and preferences	2.97 ± 0.42
Dimension 7 Self-care	2.65 ± 0.61
Dimension 8 Understanding the healthcare system	2.98 ± 0.40
Dimension 9 Capacity to process health information	3.91 ± 0.46
Dimension 10 Active engagement with HCPs	3.94 ± 0.46

### 3.5. Analysis of factors associated with cancer health literacy among caregivers

#### 3.5.1. Univariate analysis of cancer health literacy.

Univariate analysis identified significant associations between caregivers’ cancer health literacy and multiple variables (*P* < 0.05). Sociodemographic factors (gender, age, education level, occupation, per capita monthly household income, and medical insurance type), health-related behaviors (smoking, alcohol consumption), and perceived health status (self-rated health over the past week, self-assessed cancer risk) demonstrated statistical significance. Additionally, kinship to patients (e.g., spouse vs. adult child) influenced literacy levels. Complete univariate results are presented in Table 5.

**Table 5:** Univariate analysis of cancer health literacy scores among lung cancer caregivers (n = 198).

variables	n(%)	scores	t/F	*P*
Gender			t = 3.335	0.001
Male	84(42.42)	15.48 ± 3.00		
Female	114(57.58)	13.94 ± 3.34		
Age			F = 4.638	0.011
<40	66(33.33)	14.89 ± 2.66		
40-59	94(47.48)	14.96 ± 3.01		
≥60	38(19.19)	13.16 ± 4.46		
Degree of education			t = 5.139	< 0.001
High school diploma or above	113(57.07)	15.62 ± 2.48		
Middle school diploma or below	85(42.93)	13.22 ± 3.72		
marriage			t = 0.584	0.560
single	35(17.68)	14.89 ± 2.62		
married	163(82.32)	14.53 ± 3.42		
occupation			F = 19.122	< 0.001
farmer	33(16.67)	12.27 ± 4.50		
jobless	36(18.18)	13.33 ± 3.30		
staff	129(65.15)	15.53 ± 2.42		
Retirement or not			t = −1.737	0.092
Yes	18(9.09)	15.33 ± 1.68		
No	180(90.91)	14.52 ± 3.40		
Per capita monthly income of family			F = 9.845	< 0.001
<2101	74(37.38)	13.31 ± 3.85		
2101-5000	64(32.32)	15.47 ± 2.64		
>5000	60(30.30)	15.23 ± 2.64		
Medical insurance			t = 14.848	< 0.001
Urban medical insurance	82(41.62)	15.63 ± 2.35		
Rural medical insurance	115(58.38)	13.86 ± 3.66		
BMI			F = 0.561	0.571
< 18.5	9(4.55)	14.56 ± 2.30		
18.5-23.9	106(53.54)	14.37 ± 3.53		
≥24	83(41.91)	14.88 ± 3.06		
health self-assessment			t = 1.988	0.048
bad/normal	77(38.39)	14.01 ± 3.54		
good	121(61.11)	14.96 ± 3.08		
smoking			t = −3.532	0.001
Yes	65(32.83)	15.74 ± 3.29		
No	133(67.17)	14.03 ± 3.15		
Drinking			t = −4.141	< 0.001
Yes	73(36.87)	15.81 ± 3.20		
No	125(63.13)	13.88 ± 3.14		
Self-assessment of cancer risk			t = 5.276	< 0.001
Yes	99(50.00)	15.75 ± 2.72		
No	99(50.00)	13.43 ± 3.41		
Relationship with patients			F = 3.439	0.018
parents	26(13.13)	14.08 ± 3.50		
spouse	76(38.38)	13.82 ± 3.78		
children	63(31.82)	15.29 ± 2.67		
others	33(16.67)	15.25 ± 2.55		

#### 3.5.2. The correlation between cancer prevention literacy and health literacy.

Correlation analysis revealed statistically significant associations between caregivers’ total health literacy scores and the following dimensions (*P* < 0.05): cancer prevention awareness domains (preventive awareness, early detection awareness, early diagnosis awareness, and early treatment awareness), cancer-related knowledge needs, and total cancer prevention literacy scores. Detailed correlation coefficients and significance levels are presented in Table 6.

**Table 6:** Correlation between cancer prevention and treatment literacy dimensions and health literacy (HLCS-C) among caregivers (n = 198).

Variables	Dimension 1	Dimension 2	Dimension 3	Dimension 4	Dimension 5	Dimension 6	Dimension 7	Dimension 8	Dimension 9	Dimension 10	HLCS-C
Preventive awareness	0.458^**^	0.344^**^	0.232^**^	0.308^**^	0.314^**^	0.271^**^	0.444^**^	0.306^**^	0.300^**^	0.404^**^	0.472^**^
Early detection awareness	0.400	0.261^**^	0.111	0.159^*^	0.229^**^	0.245^**^	0.416^**^	0.191^**^	0.311^**^	0.284^**^	0.370^**^
Early diagnosis awareness	0.159^*^	0.113	0.074	0.161^*^	0.095	0.153^*^	0.228^**^	0.080	0.127	0.202^**^	0.194^**^
Early treatment awareness	0.057	0.143^*^	0.146^*^	0.106	0.125	0.188^**^	0.179^*^	0.085	0.044	0.176^*^	0.171^*^
Cancer-related knowledge needs	0.482^**^	0.346^**^	0.103	0.091	0.253^**^	0.189^**^	0.435^**^	0.132	0.292^**^	0.329^**^	0.380^**^
Total cancer prevention literacy scores	0.495^**^	0.373^**^	0.210^**^	0.268^**^	0.321^**^	0.310^**^	0.515^**^	0.269^**^	0.339^**^	0.426^**^	0.495^**^

*P < 0.05; ** *P <* 0.01

#### 3.5.3. Multivariable linear regression analysis of cancer health literacy.

Multivariable linear regression was employed to identify factors independently associated with caregivers’ cancer health literacy. Categorical variables were entered into the model using dummy coding. The reference categories were: Age ≥ 60; Education level: middle school diploma or below; Occupation: farmers/unemployed; Health self-assessment: bad/normal; Smoking: no; Drinking: no; Self-assessment of cancer risk: no. Variables that showed statistical significance (P < 0.05) in the univariate analyses were entered simultaneously into the regression model (forced entry method). A p-value of less than 0.05 was considered statistically significant.

The model showed excellent fit (F(12,185)=14.822, *P* < 0.001), explaining 40.9% of variance (R² = 0.409, adjusted R² = 0.360). Categorical variables were appropriately dummy-coded prior to analysis (see Table 7 for complete coding scheme and regression coefficients).

**Table 7:** Multivariable linear regression analysis of factors associated with cancer health literacy among lung cancer caregivers (n = 198).

						95.0% CI		Collinearity diagnosis	
Variables	B	SE	β	t	Sig.	lower limit	upper limit	tolerance	ViF
constant									
Age									
40-60	1.227	0.385	0.187	3.189	0.002	0.468	1.986	0.947	1.055
Degree of education									
High school diploma or above	1.385	0.498	0.209	2.784	0.006	0.302	2.366	0.577	1.734
occupation									
staff	1.162	0.542	0.169	2.144	0.023	0.093	2.232	0.524	1.908
health self-assessment									
good	1.253	0.406	0.186	3.091	0.002	0.453	2.054	0.898	1.113
smoking	1.222	0.486	0.175	2.514	0.013	0.263	2.181	0.674	1.484
drinking	1.106	0.470	0.163	2.412	0.017	0.179	2.033	0.683	1.463
Self-assessment of cancer risk	1.874	0.419	0.286	4.274	<0.001	1.048	2.700	0.802	1.247

Regression coefficient findings suggest that caregivers between 40 and 60 years old exhibit higher cancer health literacy than those in other age groups; those with higher school degree demonstrate greater health literacy than those with other educational levels; compared to farmers and the unemployed, employees exhibit higher cancer health literacy; caregivers who self-report good health have higher cancer health literacy than those who self-report poor health within the past week; individuals with habits of smoking or drinking exhibit higher health literacy than those who do not smoke or consume alcohol; patients who self-report a risk of cancer have higher cancer health literacy than those who do not perceive any cancer risk.

## 4. Discussion

### 4.1. Status of cancer health literacy among caregivers of cancer patients

As a major public health concern in China, cancer imposes substantial disease and economic burdens. Health education remains a cornerstone strategy for cancer prevention and control. In our cohort of 198 lung cancer patient caregivers, only 48.48% (n = 96) demonstrated adequate cancer prevention literacy – significantly lower than the 56.97% prevalence reported among urban residents [16]. This disparity highlights caregivers as a vulnerable population requiring targeted interventions.

Health literacy is influenced by a combination of individual, social, and environmental factors. Improving health literacy requires coordinated efforts in health education, environmental support, and policy measures to encourage individuals to adopt health behaviors related to cancer prevention and treatment. Caregivers of cancer patients often face significant economic and long-term care burdens, which can lead to prolonged physical and mental stress, adversely affecting their health outcomes and reducing their enthusiasm and compliance with caregiving responsibilities [17]. Studies have shown that an individual’s health literacy directly impacts health outcomes [18]. Therefore, enhancing caregivers’ awareness of cancer-related knowledge and their ability to utilize healthcare resources is crucial. This can improve their clinical decision-making regarding treatment options, pain management, life extension, and quality of life for cancer patients, ultimately boosting treatment compliance and cancer health literacy.

### 4.2. Factors associated with cancer health literacy among caregivers

#### 4.2.1. Age.

While our findings align with Nilsen et al. [19] regarding higher health literacy in middle-aged caregivers, they contrast with studies in Western contexts where younger caregivers, being more digitally native, often exhibit greater e-health literacy [20]. One possible explanation for this discrepancy may involve cultural differences in caregiving roles, the primary sources of health information (digital vs. traditional), and the age distribution of primary caregivers within families in China compared with Western countries. It is possible that the stability in careers and family lives during middle age, as well as proficiency in using various media platforms to access health information, contributes to the higher literacy levels observed in this group. In contrast, caregivers over 60 had lower literacy levels in our sample, which may be related to declining physical fitness, learning ability, and adaptability to new media. Older caregivers may rely more on familiar sources and have less exposure to new information [21]. Additionally, although online health information is widely available, information overload and misinformation may present particular challenges for elderly caregivers in identifying reliable resources. Taken together, these findings suggest that elderly caregivers may represent a vulnerable group in terms of cancer prevention literacy. Future educational interventions could consider targeting this population through multiple channels, though further research is needed to determine the most effective approaches.

#### 4.2.2. Education level.

Caregivers with a high school education or above scored higher in cancer health literacy (15.62 ± 2.48), consistent with findings by Lim [22], Richter [23], and Teteh [24]. Higher education levels are associated with better attention, comprehension, and analytical skills regarding health information, as well as improved communication with healthcare providers. In contrast, caregivers with lower education levels, often from rural areas, face barriers in accessing health information and tend to have outdated health behaviors and attitudes [25]. Therefore, using accessible methods (e.g., combining text and visuals) may be beneficial for promoting cancer prevention knowledge among low-educated caregivers.

#### 4.2.3. Occupation.

Caregivers employed as workers or employees scored higher in cancer health literacy (15.53 ± 2.42) compared to farmers and the unemployed, consistent with studies by Magallanes [26] and Shan Y [27]. Employed individuals often have greater exposure to multicultural environments and health resources, including regular health check-ups and education programs provided by employers. In contrast, farmers and the unemployed often face economic constraints and limited access to health information, resulting in lower health literacy [28]. Future efforts could consider disseminating cancer prevention knowledge to low-income populations and providing tailored health guidance.

#### 4.2.4. Self-rated health status.

Caregivers who self-rated their health as good in the past week scored higher in cancer health literacy (14.96 ± 3.08). This finding aligns with established theories positing health literacy as a social determinant of health, where better subjective health is often associated with greater capacity to access, understand, and apply health information [29]. Individuals with positive self-rated health likely possess more cognitive and psychological resources to engage with complex cancer-related knowledge. However, the caregiver context may present a unique paradox. While our results show a positive correlation, other studies focusing on caregivers have noted that those deeply immersed in intensive caregiving roles might report poorer self-rated health due to stress and burnout, which could subsequently impair their health literacy acquisition [30]. The discrepancy may be explained by the timing of our assessment (within one week) and the possibility that caregivers who maintain good self-rated health despite their role represent a particularly resilient subgroup with proactive health management skills. Therefore, the relationship between self-rated health and health literacy in caregivers might be bidirectional and influenced by caregiving intensity and personal resilience, warranting further longitudinal investigation.

#### 4.2.5. Smoking and alcohol consumption.

Interestingly, in our sample, caregivers who smoked or drank alcohol had higher cancer health literacy scores compared with those who did not—a finding that contrasts with Zhang et al. [31], who reported lower preventive awareness among smokers in the general population. One possible interpretation of this finding is the “teachable moment” hypothesis, which suggests that personal risk behaviors might prompt engagement with health information when caring for a lung cancer patient. Smoking and alcohol consumption are recognized risk factors for various cancers [32,33]; for example, smoking has been estimated to account for over 80% of male lung cancer deaths [34]. Tobacco smoke contains numerous carcinogens, and alcohol metabolites such as acetaldehyde have been linked to cancer risk [35,36]. It is possible that caregivers with these habits may seek medical attention more frequently and thus have greater exposure to cancer-related knowledge, which could contribute to the higher health literacy scores observed in this group. However, despite apparent awareness of the risks, many caregivers in our sample did not report adopting preventive behaviors. Future research is needed to explore the potential gap between awareness and action in this population.

#### 4.2.6. Self-assessment of cancer risk.

Caregivers who perceived themselves at risk of cancer scored higher in cancer health literacy (15.75 ± 2.72). Most caregivers in this study were first-degree relatives (FDRs) of cancer patients, who are more aware of their cancer risks and engage in proactive risk management behaviors, such as seeking health information from various sources [37]. It may be helpful for healthcare providers to guide these caregivers in adopting positive cancer prevention measures while avoiding excessive anxiety or over-prevention [38].

### 4.3. Study limitations

This study has several limitations. First, the use of convenience sampling from a single tertiary hospital in Sichuan Province restricts the generalizability of the findings. The results are primarily descriptive of the 198 participants in this specific setting and cannot be assumed to represent all lung cancer caregivers in China or other populations. Second, due to cultural differences in cancer health literacy across other countries and cultures, the findings of this study are not directly transferable to populations in other countries and cultural contexts.

## 5. Clinical Implications

This study suggests the potential importance of addressing health literacy among cancer caregivers, emphasizing its role in improving patient outcomes and caregiver well-being.

Key factors associated with caregivers’ cancer health literacy were identified, including age, education level, occupation, self-rated health status, smoking habits, alcohol consumption, and perceived cancer risk. These findings offer preliminary insights that may inform targeted interventions.

The study suggests that enhancing cancer literacy among caregivers could be considered as a component of public health promotion. Tailored strategies addressing the identified associated factors may help support this population.

## 6. Conclusion

This study investigated the cancer health literacy of 198 caregivers of lung cancer patients from a single tertiary hospital in Sichuan Province, revealing that only 96 (48.48%) in this sample had adequate literacy levels. Factors found to be associated with health literacy in this cohort included age, education level, occupation, self-rated health status, smoking, alcohol consumption, and self-assessment of cancer risk. Cancer is a critical public health issue, and early prevention and treatment are essential to reducing its incidence and improving survival rates. As primary decision-makers and caregivers for cancer patients, improving caregivers’ cancer health literacy may be an important consideration. Our findings suggest that tailored anti-cancer education could be considered based on individual characteristics, with a focus on older and less-educated caregivers. Encouraging early cancer detection, screening, and lifestyle management represents a potential avenue for further research and intervention development.

## Supporting information

S1 Data S1 Data anonymized. (XLSX)
