# Supplementary material for: The current status of health literacy among caregivers of lung cancer patients:A cross-sectional survey
Source: PLoS One. 2026 Jul 23;21(7):e0345901. doi: 10.1371/journal.pone.0345901 (PMC13395362; doi:10.1371/journal.pone.0345901)
Supplement: Attachment — Submitted filename: Response to Reviewers.docx [file pone.0345901.s004.docx]

Dear Academic Editor and Reviewers,

Thank you for your valuable feedback and constructive suggestions on our manuscript titled “The current status of health literacy among caregivers of lung cancer patients: a cross-sectional survey” (Manuscript ID: PONE-D-25-25056). We have carefully considered all comments and have revised the manuscript accordingly. Below is our point-by-point response to the comments.

1. Style requirements: We have reformatted the manuscript according to the PLOS ONE template.

2. Grant information consistency: The grant number has been unified as “2021KFKT011” throughout the manuscript.

3.Funding statement placement: All funding-related text has been removed from the Acknowledgments section and is only present in the Funding Statement.The funders had no role in study design, data collection and analysis, decision to publish, or preparation of the manuscript.

4.Data availability statement: A Data Availability Statement has been added to the end of the manuscript.

5.Supporting information captions: Captions for supporting files have been included where applicable.

6.Reference review: All references have been checked for completeness and correctness; no retracted articles were cited.

Reviewer 1:

（1）Table titles completed: We have expanded and clarified all table titles to include variables, sample size, and statistical measures.

（2）“Sex” changed to “Gender”: In Table 5, the column heading has been changed from “Sex” to “Gender.”

（3）Age categories adjusted: Age groups in Table 5 have been revised to non-overlapping intervals: <40, 40–59, and ≥60.

Reviewer 2:

（1）Introduction strengthened: The final paragraph of the Introduction now explicitly states the necessity and clinical significance of this study.

（2）Discussion expanded: We have incorporated comparisons with existing literature in the Discussion section, highlighting both consistent and contradictory findings, along with potential explanations.

We believe these revisions have significantly improved the manuscript and hope it now meets the publication standards of PLOS ONE. Thank you again for your time and consideration.

Sincerely,

Lei lei

On behalf of all authors
