# Supplementary material for: The current status of health literacy among caregivers of lung cancer patients:A cross-sectional survey
Source: PLoS One. 2026 Jul 23;21(7):e0345901. doi: 10.1371/journal.pone.0345901 (PMC13395362; doi:10.1371/journal.pone.0345901)
Supplement: Attachment — Submitted filename: plos one 1.5.1404.docx [file pone.0345901.s002.docx]

Thanks to the authors for choosing the important topic of health literacy.

The results section should be reviewed by a statistical reviewer.

But a few points are essential.

Complete the table titles.

Use the word gender instead of sex (Table 5).

The age categories overlap at age 40 (Table 5).
