# Supplementary material for: The current status of health literacy among caregivers of lung cancer patients:A cross-sectional survey
Source: PLoS One. 2026 Jul 23;21(7):e0345901. doi: 10.1371/journal.pone.0345901 (PMC13395362; doi:10.1371/journal.pone.0345901)
Supplement: Attachment — Submitted filename: Response to Reviewers2.docx [file pone.0345901.s005.docx]

Dear Editor and Reviewers,

We would like to express our sincere gratitude for your thoughtful and constructive comments on our manuscript (Manuscript ID: PONE-D-25-25056). **All revisions in the manuscript have been highlighted in blue font for easy identification.**These comments have been invaluable in improving the quality of our work. We have carefully addressed each point raised by the reviewers and have revised the manuscript accordingly. Below we provide a point-by-point response.

**Response to Reviewer #2**

Comment: Your commentary on the role of health literacy as a social determinant of health makes sense, and your explanation of the possibility that this relationship is bidirectional in the context of caregivers adds to the richness of the discussion. It is also valuable to point out the role of resilience and caregiving intensity as potential moderating variables.

Response: We thank the reviewer for these positive and encouraging remarks. No specific revision was requested. We have retained the relevant content in the Discussion section (section 4.2.4) as it was positively received.

**Response to Reviewer #3**

Comment 1: The manuscript presents research aims but does not state a formal hypothesis (null or alternative) nor define a primary endpoint. This is a fundamental methodological limitation.

Response: We apologize for this omission. We have now explicitly stated the primary hypothesis and defined the primary and secondary endpoints in the Abstract (Aim), the end of the Introduction, and in the Methods section (2.2.2 and 2.2.3). Specifically:

Primary hypothesis: Caregivers of lung cancer patients would demonstrate suboptimal cancer health literacy.

Primary endpoint: Total score on the Chinese Cancer Health Literacy Scale (C-CHLS).

Secondary endpoints: Scores on the Health Literacy of Caregivers Scale-Cancer (HLCS-C) and the identification of factors associated with caregivers’ health literacy.

Comment 2: You administered two separate scales. Which one is the primary outcome? What are your secondary outcomes?

Response: As noted above, we have now clearly designated the C-CHLS total score as the primary endpoint and the HLCS-C scores as secondary endpoints. Corresponding statements have been added at the end of sections 2.2.2 and 2.2.3.

Comment 3: Without a pre-specified hypothesis and primary endpoint, and without adjustment for multiple testing, the reported p-values are difficult to interpret.

Response: We thank the reviewer for raising this important statistical issue. In response, we have:

1.Clearly stated the exploratory and hypothesis-generating nature of the study in the Abstract, Introduction, and Methods.

2.Given the exploratory design, we now explicitly state that no adjustment for multiple comparisons was applied in the univariate analyses, and findings should be considered hypothesis-generating. Only variables with p < 0.05 were entered into the multivariable regression model (Methods 2.5).Clarified that the multivariable regression was hypothesis-driven and used p < 0.05 as the significance threshold.

These revisions ensure that the statistical approach is consistent with the exploratory nature of the study and transparently reported.

Comment 4: Convenience sampling is used. The sample is not representative of any defined target population. This has implications for generalizability and statistical inference.

Response: We fully acknowledge this limitation. We have:

1.Added a statement in Methods 2.1 that a convenience sampling method was used.

2.Revised the Limitations section (4.3) to clearly state that the findings are primarily descriptive of the 198 participants from a single tertiary hospital in Sichuan Province and cannot be assumed to represent all lung cancer caregivers in China or other populations.

3.Modified the Abstract Conclusion and main Conclusion to avoid overgeneralization, using phrases such as “in this sample,” “provide preliminary evidence,” and “in similar clinical settings.”

These changes address the reviewer’s concern about overreach and ensure that conclusions are appropriately circumscribed.

Comment 5: The manuscript does not clearly state that the study is exploratory and hypothesis-generating.

Response: We have now explicitly characterized the study as “exploratory and hypothesis-generating” in the Abstract (Aim), Introduction (final paragraph), and Methods (Data Analysis section).

Comment 6: The manuscript should avoid causal language in the interpretation of results (Discussion and Conclusion sections). Findings are associative and should be framed as such.

Response: We have systematically revised the language throughout the Discussion and Conclusion to avoid causal implications.

We have also revised section 4.1 (Status of Cancer Health Literacy) extensively to remove causal phrasing.

Minor comment 1: Multivariable regression analysis is not fully described. Please define dummy coding and reference categories, specify the model-building strategy, and revise terminology from “multivariate” to “multivariable”.

Response: We have:

1.Corrected all instances of “multivariate” to “multivariable” throughout the manuscript (Abstract, Methods, Results headings, Table 7).

2.Added detailed dummy coding information in Methods 2.5 and Results 3.5.3, including reference categories for each categorical variable.

3.Specified the model-building strategy (forced entry method) in the revised Methods section.

Minor comment 2: Correct inconsistencies in age groups (e.g., Table 5 shows “40-60” and “>60” rather than “40-59” and “≥60”).

Response: We have corrected the age group labels in Table 5 to match Table 1: now “<40”, “40-59”, and “≥60”. All inconsistencies have been eliminated.

We believe that these revisions have substantially improved the manuscript and have addressed all concerns raised by the reviewers. We are grateful for the reviewers’ time and expertise, and we hope that the revised manuscript is now acceptable for publication in your journal.

Should any further modifications be required, we would be happy to make them.

Yours sincerely,

Lei Lei, on behalf of all authors.
