# Supplementary material for: The current status of health literacy among caregivers of lung cancer patients:A cross-sectional survey
Source: PLoS One. 2026 Jul 23;21(7):e0345901. doi: 10.1371/journal.pone.0345901 (PMC13395362; doi:10.1371/journal.pone.0345901)
Supplement: Peer review history

# Peer review: Decision Letter 0

**DOI:** 10.1371/journal.pone.0345901.r001

**Authors:** Mojtaba Fattahi Ardakani

26 Nov 2025

PONE-D-25-25056

The current status of health literacy among caregivers of lung cancer patients�a cross-sectional survey

PLOS ONE

Dear Dr. lei,

Thank you for submitting your manuscript to PLOS ONE. After careful consideration, we feel that it has merit but does not fully meet PLOS ONE’s publication criteria as it currently stands. Therefore, we invite you to submit a revised version of the manuscript that addresses the points raised during the review process.

Please submit your revised manuscript by Jan 10 2026 11:59PM. If you will need more time than this to complete your revisions, please reply to this message or contact the journal office at plosone@plos.org. When you're ready to submit your revision, log on to https://www.editorialmanager.com/pone/ and select the 'Submissions Needing Revision' folder to locate your manuscript file.

Please include the following items when submitting your revised manuscript:

- A rebuttal letter that responds to each point raised by the academic editor and reviewer(s). You should upload this letter as a separate file labeled 'Response to Reviewers'.
- A marked-up copy of your manuscript that highlights changes made to the original version. You should upload this as a separate file labeled 'Revised Manuscript with Track Changes'.
- An unmarked version of your revised paper without tracked changes. You should upload this as a separate file labeled 'Manuscript'.

-->If you would like to make changes to your financial disclosure, please include your updated statement in your cover letter. Guidelines for resubmitting your figure files are available below the reviewer comments at the end of this letter.

If applicable, we recommend that you deposit your laboratory protocols in protocols.io to enhance the reproducibility of your results. Protocols.io assigns your protocol its own identifier (DOI) so that it can be cited independently in the future. For instructions see: https://journals.plos.org/plosone/s/submission-guidelines#loc-laboratory-protocols. Additionally, PLOS ONE offers an option for publishing peer-reviewed Lab Protocol articles, which describe protocols hosted on protocols.io. Read more information on sharing protocols at https://plos.org/protocols?utm_medium=editorial-email&utm_source=authorletters&utm_campaign=protocols.

We look forward to receiving your revised manuscript.

Kind regards,

Mojtaba Fattahi Ardakani

Academic Editor

PLOS ONE

Journal Requirements:

When submitting your revision, we need you to address these additional requirements.

1. Please ensure that your manuscript meets PLOS ONE's style requirements, including those for file naming. The PLOS ONE style templates can be found at

https://journals.plos.org/plosone/s/file?id=wjVg/PLOSOne_formatting_sample_main_body.pdf and

https://journals.plos.org/plosone/s/file?id=ba62/PLOSOne_formatting_sample_title_authors_affiliations.pdf

2. We note that the grant information you provided in the ‘Funding Information’ and ‘Financial Disclosure’ sections do not match.

When you resubmit, please ensure that you provide the correct grant numbers for the awards you received for your study in the ‘Funding Information’ section.

3. Thank you for stating the following financial disclosure:

“The open Project of State Key Laboratory of Ultrasonic Medical Engineering in 2021”

Please state what role the funders took in the study. If the funders had no role, please state: "The funders had no role in study design, data collection and analysis, decision to publish, or preparation of the manuscript."

If this statement is not correct you must amend it as needed.

Please include this amended Role of Funder statement in your cover letter; we will change the online submission form on your behalf.

4. Thank you for stating the following in the Acknowledgments Section of your manuscript:

“This work was supported by The open Project of State Key Laboratory of Ultrasonic Medical Engineering in 2021�Project No. 2021KFKT010.”

We note that you have provided funding information that is currently declared in your Funding Statement. However, funding information should not appear in the Acknowledgments section or other areas of your manuscript. We will only publish funding information present in the Funding Statement section of the online submission form.

Please remove any funding-related text from the manuscript and let us know how you would like to update your Funding Statement. Currently, your Funding Statement reads as follows:

“The open Project of State Key Laboratory of Ultrasonic Medical Engineering in 2021”

Please include your amended statements within your cover letter; we will change the online submission form on your behalf.

5. When completing the data availability statement of the submission form, you indicated that you will make your data available on acceptance. We strongly recommend all authors decide on a data sharing plan before acceptance, as the process can be lengthy and hold up publication timelines. Please note that, though access restrictions are acceptable now, your entire data will need to be made freely accessible if your manuscript is accepted for publication. This policy applies to all data except where public deposition would breach compliance with the protocol approved by your research ethics board. If you are unable to adhere to our open data policy, please kindly revise your statement to explain your reasoning and we will seek the editor's input on an exemption. Please be assured that, once you have provided your new statement, the assessment of your exemption will not hold up the peer review process.

6.  Please include captions for your Supporting Information files at the end of your manuscript, and update any in-text citations to match accordingly. Please see our Supporting Information guidelines for more information: http://journals.plos.org/plosone/s/supporting-information.

7. If the reviewer comments include a recommendation to cite specific previously published works, please review and evaluate these publications to determine whether they are relevant and should be cited. There is no requirement to cite these works unless the editor has indicated otherwise.

8. Please review your reference list to ensure that it is complete and correct. If you have cited papers that have been retracted, please include the rationale for doing so in the manuscript text, or remove these references and replace them with relevant current references. Any changes to the reference list should be mentioned in the rebuttal letter that accompanies your revised manuscript. If you need to cite a retracted article, indicate the article’s retracted status in the References list and also include a citation and full reference for the retraction notice.

[Note: HTML markup is below. Please do not edit.]

Reviewers' comments:

Reviewer's Responses to Questions

**Comments to the Author**

1. Is the manuscript technically sound, and do the data support the conclusions?

The manuscript must describe a technically sound piece of scientific research with data that supports the conclusions. Experiments must have been conducted rigorously, with appropriate controls, replication, and sample sizes. The conclusions must be drawn appropriately based on the data presented.

Reviewer #1: Yes

Reviewer #2: Yes

**********

2. Has the statistical analysis been performed appropriately and rigorously?

Reviewer #1: I Don't Know

Reviewer #2: I Don't Know

**********

3. Have the authors made all data underlying the findings in their manuscript fully available?

The PLOS Data policy requires authors to make all data underlying the findings described in their manuscript fully available without restriction, with rare exception (please refer to the Data Availability Statement in the manuscript PDF file). The data should be provided as part of the manuscript or its supporting information, or deposited to a public repository. For example, in addition to summary statistics, the data points behind means, medians and variance measures should be available. If there are restrictions on publicly sharing data—e.g. participant privacy or use of data from a third party—those must be specified.

Reviewer #1: Yes

Reviewer #2: Yes

**********

4. Is the manuscript presented in an intelligible fashion and written in standard English?

PLOS ONE does not copyedit accepted manuscripts, so the language in submitted articles must be clear, correct, and unambiguous. Any typographical or grammatical errors should be corrected at revision, so please note any specific errors here.

Reviewer #1: Yes

Reviewer #2: Yes

**********

5. Review Comments to the Author

Please use the space provided to explain your answers to the questions above. You may also include additional comments for the author, including concerns about dual publication, research ethics, or publication ethics. (Please upload your review as an attachment if it exceeds 20,000 characters)

Reviewer #1: Thanks to the authors for choosing the important topic of health literacy.

The results section should be reviewed by a statistical reviewer.

But a few points are essential.

Complete the table titles.

Use the word gender instead of sex (Table 5).

The age categories overlap at age 40 (Table 5).

Reviewer #2: In the last paragraph of the introduction, the necessity and importance of conducting the study should be mentioned.

Please also refer to the contradictory findings of other studies in comparison with the results of the present study in discussion

**********

6. PLOS authors have the option to publish the peer review history of their article (what does this mean?). If published, this will include your full peer review and any attached files.

If you choose “no”, your identity will remain anonymous but your review may still be made public.

**Do you want your identity to be public for this peer review?**  For information about this choice, including consent withdrawal, please see our Privacy Policy.

Reviewer #1: **Yes:** Nahid Ardian

Reviewer #2: No

**********

[NOTE: If reviewer comments were submitted as an attachment file, they will be attached to this email and accessible via the submission site. Please log into your account, locate the manuscript record, and check for the action link "View Attachments". If this link does not appear, there are no attachment files.]

To ensure your figures meet our technical requirements, please review our figure guidelines: https://journals.plos.org/plosone/s/figures

You may also use PLOS’s free figure tool, NAAS, to help you prepare publication quality figures: https://journals.plos.org/plosone/s/figures#loc-tools-for-figure-preparation.

NAAS will assess whether your figures meet our technical requirements by comparing each figure against our figure specifications.

Attachment Submitted filename: plos one 1.5.1404.docx

# Author comment: Author response to Decision Letter 1

**DOI:** 10.1371/journal.pone.0345901.r002

6 Jan 2026

Dear Academic Editor and Reviewers,

Thank you for your valuable feedback and constructive suggestions on our manuscript titled “The current status of health literacy among caregivers of lung cancer patients: a cross-sectional survey” (Manuscript ID: PONE-D-25-25056). We have carefully considered all comments and have revised the manuscript accordingly. Below is our point-by-point response to the comments.

1. Style requirements: We have reformatted the manuscript according to the PLOS ONE template.

2. Grant information consistency: The grant number has been unified as “2021KFKT011” throughout the manuscript.

3.Funding statement placement: All funding-related text has been removed from the Acknowledgments section and is only present in the Funding Statement.The funders had no role in study design, data collection and analysis, decision to publish, or preparation of the manuscript.

4.Data availability statement: A Data Availability Statement has been added to the end of the manuscript.

5.Supporting information captions: Captions for supporting files have been included where applicable.

6.Reference review: All references have been checked for completeness and correctness; no retracted articles were cited.

Reviewer 1:

�1�Table titles completed: We have expanded and clarified all table titles to include variables, sample size, and statistical measures.

�2�“Sex” changed to “Gender”: In Table 5, the column heading has been changed from “Sex” to “Gender.”

�3�Age categories adjusted: Age groups in Table 5 have been revised to non-overlapping intervals: <40, 40–59, and ≥60.

Reviewer 2:

�1�Introduction strengthened: The final paragraph of the Introduction now explicitly states the necessity and clinical significance of this study.

�2�Discussion expanded: We have incorporated comparisons with existing literature in the Discussion section, highlighting both consistent and contradictory findings, along with potential explanations.

We believe these revisions have significantly improved the manuscript and hope it now meets the publication standards of PLOS ONE. Thank you again for your time and consideration.

Sincerely,

Lei lei

On behalf of all authors

Attachment Submitted filename: Response to Reviewers.docx

# Peer review: Decision Letter 1

**DOI:** 10.1371/journal.pone.0345901.r003

**Authors:** Helen Howard

31 Mar 2026

PONE-D-25-25056R1

The current status of health literacy among caregivers of lung cancer patients�a cross-sectional survey

PLOS One

Dear Dr. lei,

Thank you for submitting your manuscript to PLOS ONE. After careful consideration, we feel that it has merit but does not fully meet PLOS ONE’s publication criteria as it currently stands. Therefore, we invite you to submit a revised version of the manuscript that addresses the points raised during the review process.

Please submit your revised manuscript by May 15 2026 11:59PM. If you will need more time than this to complete your revisions, please reply to this message or contact the journal office at plosone@plos.org. When you're ready to submit your revision, log on to https://www.editorialmanager.com/pone/ and select the 'Submissions Needing Revision' folder to locate your manuscript file.

Please include the following items when submitting your revised manuscript:

- A letter that responds to each point raised by the academic editor and reviewer(s). You should upload this letter as a separate file labeled 'Response to Reviewers'.
- A marked-up copy of your manuscript that highlights changes made to the original version. You should upload this as a separate file labeled 'Revised Manuscript with Track Changes'.
- An unmarked version of your revised paper without tracked changes. You should upload this as a separate file labeled 'Manuscript'.

If you would like to make changes to your financial disclosure, please include your updated statement in your cover letter. Guidelines for resubmitting your figure files are available below the reviewer comments at the end of this letter.

If applicable, we recommend that you deposit your laboratory protocols in protocols.io to enhance the reproducibility of your results. Protocols.io assigns your protocol its own identifier (DOI) so that it can be cited independently in the future. For instructions see: https://journals.plos.org/plosone/s/submission-guidelines#loc-laboratory-protocols. Additionally, PLOS ONE offers an option for publishing peer-reviewed Lab Protocol articles, which describe protocols hosted on protocols.io. Read more information on sharing protocols at https://plos.org/protocols?utm_medium=editorial-email&utm_source=authorletters&utm_campaign=protocols.

As the corresponding author, your ORCID iD is verified in the submission system and will appear in the published article. PLOS supports the use of ORCID, and we encourage all coauthors to register for an ORCID iD and use it as well. Please encourage your coauthors to verify their ORCID iD within the submission system before final acceptance, as unverified ORCID iDs will not appear in the published article. *Only*  the individual author can complete the verification step; PLOS staff *cannot*  verify ORCID iDs on behalf of authors.

We look forward to receiving your revised manuscript.

Kind regards,

Helen Howard

Staff Editor

PLOS One

Journal Requirements:

If the reviewer comments include a recommendation to cite specific previously published works, please review and evaluate these publications to determine whether they are relevant and should be cited. There is no requirement to cite these works unless the editor has indicated otherwise.

[Note: HTML markup is below. Please do not edit.]

Reviewers' comments:

Reviewer's Responses to Questions

**Comments to the Author**

1. If the authors have adequately addressed your comments raised in a previous round of review and you feel that this manuscript is now acceptable for publication, you may indicate that here to bypass the “Comments to the Author” section, enter your conflict of interest statement in the “Confidential to Editor” section, and submit your "Accept" recommendation.

Reviewer #2: All comments have been addressed

Reviewer #3: (No Response)

**********

2. Is the manuscript technically sound, and do the data support the conclusions?

The manuscript must describe a technically sound piece of scientific research with data that supports the conclusions. Experiments must have been conducted rigorously, with appropriate controls, replication, and sample sizes. The conclusions must be drawn appropriately based on the data presented.

Reviewer #2: Yes

Reviewer #3: (No Response)

**********

3. Has the statistical analysis been performed appropriately and rigorously?

Reviewer #2: I Don't Know

Reviewer #3: (No Response)

**********

4. Have the authors made all data underlying the findings in their manuscript fully available?

The PLOS Data policy requires authors to make all data underlying the findings described in their manuscript fully available without restriction, with rare exception (please refer to the Data Availability Statement in the manuscript PDF file). The data should be provided as part of the manuscript or its supporting information, or deposited to a public repository. For example, in addition to summary statistics, the data points behind means, medians and variance measures should be available. If there are restrictions on publicly sharing data—e.g. participant privacy or use of data from a third party—those must be specified.

Reviewer #2: Yes

Reviewer #3: (No Response)

**********

5. Is the manuscript presented in an intelligible fashion and written in standard English?

PLOS ONE does not copyedit accepted manuscripts, so the language in submitted articles must be clear, correct, and unambiguous. Any typographical or grammatical errors should be corrected at revision, so please note any specific errors here.

Reviewer #2: Yes

Reviewer #3: (No Response)

**********

6. Review Comments to the Author

Please use the space provided to explain your answers to the questions above. You may also include additional comments for the author, including concerns about dual publication, research ethics, or publication ethics. (Please upload your review as an attachment if it exceeds 20,000 characters)

Reviewer #2: Your commentary on the role of health literacy as a social determinant of health makes sense, and your explanation of the possibility that this relationship is bidirectional in the context of caregivers adds to the richness of the discussion. It is also valuable to point out the role of resilience and caregiving intensity as potential moderating variables.

Reviewer #3: Below are some comments:

1. The manuscript presents research aims but does not state a formal hypothesis (null or alternative) nor define a primary endpoint. This is a fundamental methodological limitation. Without a hypothesis, it is unclear what specific predictions the study is testing. Without a primary endpoint, it is unclear which outcome measure answers the main research question.

2. You administered two separate scales (C-CHLS, lines 130-138; and HLCS-C, lines 139-149). Which one is the primary outcome for your main analysis? What are your secondary outcomes?

3. The manuscript has univariate analysis (Table 5, lines 262 ...) examines numerous comparisons across multiple variables and outcomes. Your multivariable model (Table 7, lines 286 ...) includes seven predictors. Without a pre-specified hypothesis and primary endpoint, and without adjustment for multiple testing, the reported p-values are difficult to interpret as measures of statistical significance. Please clarify.

4. Convenience sampling is used (line 27), a non-probability sampling method. The sample is not representative of any defined target population. This has three direct implications that your manuscript does not address:

- Inferential statistics (p-values, confidence intervals, regression coefficients) assume random sampling. With convenience sampling, these inferential statistics are not technically valid for population inference.

- Your limitation statement should be revised. You state the findings are "only applicable to the Chinese population" (lines 398-400). Your findings are only applicable to the 198 individuals in your convenience sample from a single tertiary hospital in Sichuan. Even generalization to Sichuan Province is not justified.

- Your conclusions overreach. Your Abstract (lines 39-43) and Conclusion (lines 414-424) state: "underscore the urgent need for tailored educational interventions," "provide evidence for developing targeted strategies," "improve overall population health." These conclusions assume your sample represents a broader population of caregivers. Given the convenience sampling, such generalizations are overreach.

5. The manuscript does not clearly state that the study is exploratory and hypothesis-generating. This should be explicitly clarified to appropriately frame the analysis and interpretation of the results.

6. The manuscript should avoid causal language in the interpretation of results (Discussion and Conclusion sections). The findings are associative and should be framed as such, with implications presented as hypotheses for future research rather than clinical recommendations.

Minor comments:

1. The multivariable regression analysis is not fully described (Methods, lines 182-183; Results/Table 7). Please:

- Clearly define dummy coding and reference categories for all categorical variables

- Specify the model-building strategy (e.g., forced entry or selection based on univariate p-values).

- Revise terminology from “multivariate” to “multivariable” linear regression.

2. Correct all inconsistencies as outlined in the previous review, such as age groups, in Table 5 still shows "40-60" and ">60" rather than "40-59" and "≥60."

**********

7. PLOS authors have the option to publish the peer review history of their article (what does this mean?). If published, this will include your full peer review and any attached files.

If you choose “no”, your identity will remain anonymous but your review may still be made public.

**Do you want your identity to be public for this peer review?**  For information about this choice, including consent withdrawal, please see our Privacy Policy.

Reviewer #2: No

Reviewer #3: No

**********

[NOTE: If reviewer comments were submitted as an attachment file, they will be attached to this email and accessible via the submission site. Please log into your account, locate the manuscript record, and check for the action link "View Attachments". If this link does not appear, there are no attachment files.]

To ensure your figures meet our technical requirements, please review our figure guidelines: https://journals.plos.org/plosone/s/figures

You may also use PLOS’s free figure tool, NAAS, to help you prepare publication quality figures: https://journals.plos.org/plosone/s/figures#loc-tools-for-figure-preparation.

NAAS will assess whether your figures meet our technical requirements by comparing each figure against our figure specifications.

# Author comment: Author response to Decision Letter 2

**DOI:** 10.1371/journal.pone.0345901.r004

27 Apr 2026

Dear Editor and Reviewers,

We would like to express our sincere gratitude for your thoughtful and constructive comments on our manuscript (Manuscript ID: PONE-D-25-25056). All revisions in the manuscript have been highlighted in blue font for easy identification.These comments have been invaluable in improving the quality of our work. We have carefully addressed each point raised by the reviewers and have revised the manuscript accordingly. Below we provide a point-by-point response.

Response to Reviewer #2

Comment: Your commentary on the role of health literacy as a social determinant of health makes sense, and your explanation of the possibility that this relationship is bidirectional in the context of caregivers adds to the richness of the discussion. It is also valuable to point out the role of resilience and caregiving intensity as potential moderating variables.

Response: We thank the reviewer for these positive and encouraging remarks. No specific revision was requested. We have retained the relevant content in the Discussion section (section 4.2.4) as it was positively received.

Response to Reviewer #3

Comment 1: The manuscript presents research aims but does not state a formal hypothesis (null or alternative) nor define a primary endpoint. This is a fundamental methodological limitation.

Response: We apologize for this omission. We have now explicitly stated the primary hypothesis and defined the primary and secondary endpoints in the Abstract (Aim), the end of the Introduction, and in the Methods section (2.2.2 and 2.2.3). Specifically:

Primary hypothesis: Caregivers of lung cancer patients would demonstrate suboptimal cancer health literacy.

Primary endpoint: Total score on the Chinese Cancer Health Literacy Scale (C-CHLS).

Secondary endpoints: Scores on the Health Literacy of Caregivers Scale-Cancer (HLCS-C) and the identification of factors associated with caregivers’ health literacy.

Comment 2: You administered two separate scales. Which one is the primary outcome? What are your secondary outcomes?

Response: As noted above, we have now clearly designated the C-CHLS total score as the primary endpoint and the HLCS-C scores as secondary endpoints. Corresponding statements have been added at the end of sections 2.2.2 and 2.2.3.

Comment 3: Without a pre-specified hypothesis and primary endpoint, and without adjustment for multiple testing, the reported p-values are difficult to interpret.

Response: We thank the reviewer for raising this important statistical issue. In response, we have:

1.Clearly stated the exploratory and hypothesis-generating nature of the study in the Abstract, Introduction, and Methods.

2.Given the exploratory design, we now explicitly state that no adjustment for multiple comparisons was applied in the univariate analyses, and findings should be considered hypothesis-generating. Only variables with p < 0.05 were entered into the multivariable regression model (Methods 2.5).Clarified that the multivariable regression was hypothesis-driven and used p < 0.05 as the significance threshold.

These revisions ensure that the statistical approach is consistent with the exploratory nature of the study and transparently reported.

Comment 4: Convenience sampling is used. The sample is not representative of any defined target population. This has implications for generalizability and statistical inference.

Response: We fully acknowledge this limitation. We have:

1.Added a statement in Methods 2.1 that a convenience sampling method was used.

2.Revised the Limitations section (4.3) to clearly state that the findings are primarily descriptive of the 198 participants from a single tertiary hospital in Sichuan Province and cannot be assumed to represent all lung cancer caregivers in China or other populations.

3.Modified the Abstract Conclusion and main Conclusion to avoid overgeneralization, using phrases such as “in this sample,” “provide preliminary evidence,” and “in similar clinical settings.”

These changes address the reviewer’s concern about overreach and ensure that conclusions are appropriately circumscribed.

Comment 5: The manuscript does not clearly state that the study is exploratory and hypothesis-generating.

Response: We have now explicitly characterized the study as “exploratory and hypothesis-generating” in the Abstract (Aim), Introduction (final paragraph), and Methods (Data Analysis section).

Comment 6: The manuscript should avoid causal language in the interpretation of results (Discussion and Conclusion sections). Findings are associative and should be framed as such.

Response: We have systematically revised the language throughout the Discussion and Conclusion to avoid causal implications.

We have also revised section 4.1 (Status of Cancer Health Literacy) extensively to remove causal phrasing.

Minor comment 1: Multivariable regression analysis is not fully described. Please define dummy coding and reference categories, specify the model-building strategy, and revise terminology from “multivariate” to “multivariable”.

Response: We have:

1.Corrected all instances of “multivariate” to “multivariable” throughout the manuscript (Abstract, Methods, Results headings, Table 7).

2.Added detailed dummy coding information in Methods 2.5 and Results 3.5.3, including reference categories for each categorical variable.

3.Specified the model-building strategy (forced entry method) in the revised Methods section.

Minor comment 2: Correct inconsistencies in age groups (e.g., Table 5 shows “40-60” and “>60” rather than “40-59” and “≥60”).

Response: We have corrected the age group labels in Table 5 to match Table 1: now “<40”, “40-59”, and “≥60”. All inconsistencies have been eliminated.

We believe that these revisions have substantially improved the manuscript and have addressed all concerns raised by the reviewers. We are grateful for the reviewers’ time and expertise, and we hope that the revised manuscript is now acceptable for publication in your journal.

Should any further modifications be required, we would be happy to make them.

Yours sincerely,

Lei Lei, on behalf of all authors.

Attachment Submitted filename: Response to Reviewers2.docx

# Peer review: Decision Letter 2

**DOI:** 10.1371/journal.pone.0345901.r005

**Authors:** Jie Yang

25 May 2026

The current status of health literacy among caregivers of lung cancer patients�a cross-sectional survey

PONE-D-25-25056R2

Dear Dr. lei,

We’re pleased to inform you that your manuscript has been judged scientifically suitable for publication and will be formally accepted for publication once it meets all outstanding technical requirements.

Within one week, you’ll receive an e-mail detailing the required amendments. When these have been addressed, you’ll receive a formal acceptance letter and your manuscript will be scheduled for publication.

An invoice will be generated when your article is formally accepted. Please note, if your institution has a publishing partnership with PLOS and your article meets the relevant criteria, all or part of your publication costs will be covered. Please make sure your user information is up-to-date by logging into Editorial Manager at Editorial Manager® and clicking the ‘Update My Information' link at the top of the page. For questions related to billing, please contact billing support.

If your institution or institutions have a press office, please notify them about your upcoming paper to help maximize its impact. If they’ll be preparing press materials, please inform our press team as soon as possible -- no later than 48 hours after receiving the formal acceptance. Your manuscript will remain under strict press embargo until 2 pm Eastern Time on the date of publication. For more information, please contact onepress@plos.org.

Kind regards,

Jie Yang, M.D.

Guest Editor

PLOS One

Additional Editor Comments (optional):

Thanks for the authors' efforts to comprehensively improve your manuscript according to editor's and reviewers' comments. I am pleased to inform you that your paper can be accepted for publication now. Thanks for the chance to assess your work. Additionally, many thanks for all the reviewers' precious inputs.

Reviewers' comments:

Reviewer's Responses to Questions

**Comments to the Author**

1. If the authors have adequately addressed your comments raised in a previous round of review and you feel that this manuscript is now acceptable for publication, you may indicate that here to bypass the “Comments to the Author” section, enter your conflict of interest statement in the “Confidential to Editor” section, and submit your "Accept" recommendation.

Reviewer #3: All comments have been addressed

**********

2. Is the manuscript technically sound, and do the data support the conclusions?

The manuscript must describe a technically sound piece of scientific research with data that supports the conclusions. Experiments must have been conducted rigorously, with appropriate controls, replication, and sample sizes. The conclusions must be drawn appropriately based on the data presented.

Reviewer #3: (No Response)

**********

3. Has the statistical analysis been performed appropriately and rigorously?

Reviewer #3: (No Response)

**********

4. Have the authors made all data underlying the findings in their manuscript fully available?

The PLOS Data policy requires authors to make all data underlying the findings described in their manuscript fully available without restriction, with rare exception (please refer to the Data Availability Statement in the manuscript PDF file). The data should be provided as part of the manuscript or its supporting information, or deposited to a public repository. For example, in addition to summary statistics, the data points behind means, medians and variance measures should be available. If there are restrictions on publicly sharing data—e.g. participant privacy or use of data from a third party—those must be specified.

Reviewer #3: (No Response)

**********

5. Is the manuscript presented in an intelligible fashion and written in standard English?

PLOS ONE does not copyedit accepted manuscripts, so the language in submitted articles must be clear, correct, and unambiguous. Any typographical or grammatical errors should be corrected at revision, so please note any specific errors here.

Reviewer #3: (No Response)

**********

6. Review Comments to the Author

Please use the space provided to explain your answers to the questions above. You may also include additional comments for the author, including concerns about dual publication, research ethics, or publication ethics. (Please upload your review as an attachment if it exceeds 20,000 characters)

Reviewer #3: (No Response)

**********

7. PLOS authors have the option to publish the peer review history of their article (what does this mean?). If published, this will include your full peer review and any attached files.

If you choose “no”, your identity will remain anonymous but your review may still be made public.

**Do you want your identity to be public for this peer review?**  For information about this choice, including consent withdrawal, please see our Privacy Policy.

Reviewer #3: No

**********

# Editor report: Acceptance letter

**DOI:** 10.1371/journal.pone.0345901.r006

**Authors:** Jie Yang

PONE-D-25-25056R2

PLOS One

Dear Dr. lei,

I'm pleased to inform you that your manuscript has been deemed suitable for publication in PLOS One. Congratulations! Your manuscript is now being handed over to our production team.

At this stage, our production department will prepare your paper for publication. This includes ensuring the following:

* All references, tables, and figures are properly cited

* All relevant supporting information is included in the manuscript submission,

* There are no issues that prevent the paper from being properly typeset

You will receive further instructions from the production team, including instructions on how to review your proof when it is ready. Please keep in mind that we are working through a large volume of accepted articles, so please give us a few days to review your paper and let you know the next and final steps.

Lastly, if your institution or institutions have a press office, please let them know about your upcoming paper now to help maximize its impact. If they'll be preparing press materials, please inform our press team within the next 48 hours. Your manuscript will remain under strict press embargo until 2 pm Eastern Time on the date of publication. For more information, please contact onepress@plos.org.

You will receive an invoice from PLOS for your publication fee after your manuscript has reached the completed accept phase. If you receive an email requesting payment before acceptance or for any other service, this may be a phishing scheme. Learn how to identify phishing emails and protect your accounts at https://explore.plos.org/phishing.

If we can help with anything else, please email us at customercare@plos.org.

Thank you for submitting your work to PLOS ONE and supporting open access.

Kind regards,

PLOS ONE Editorial Office Staff

on behalf of

Dr. Jie Yang

Guest Editor

PLOS One
